# Detection of suspicious interactions of spiking covariates in methylation data

**DOI:** 10.1186/s12859-020-3364-6

**Published:** 2020-01-30

**Authors:** Miriam Sieg, Gesa Richter, Arne S. Schaefer, Jochen Kruppa

**Affiliations:** 1 Charité - University Medicine, corporate member of Freie Universität Berlin, Humboldt-Universität zu Berlin, and Berlin Institute of Health, Institute of Biometry and Clinical Epidemiology, Charitéplatz 1, Berlin, 10117 Germany; 2 Berlin Institute of Health (BIH), Anna-Louisa-Karsch-Strane 2, Berlin, 10178 Germany; 3 Department of Periodontology and Synoptic Dentistry, Institute of Dental, Oral and Maxillary Medicine, Charité - University Medicine, Charitéplatz 1, Berlin, 10117 Germany

**Keywords:** Spike at zero, Methylation, Outlier detection, Epigenetic, High dimensional data

## Abstract

**Background:**

In methylation analyses like epigenome-wide association studies, a high amount of biomarkers is tested for an association between the measured continuous outcome and different covariates. In the case of a continuous covariate like smoking pack years (SPY), a measure of lifetime exposure to tobacco toxins, a spike at zero can occur. Hence, all non-smokers are generating a peak at zero, while the smoking patients are distributed over the other SPY values. Additionally, the spike might also occur on the right side of the covariate distribution, if a category “heavy smoker” is designed. Here, we will focus on methylation data with a spike at the left or the right of the distribution of a continuous covariate. After the methylation data is generated, analysis is usually performed by preprocessing, quality control, and determination of differentially methylated sites, often performed in pipeline fashion. Hence, the data is processed in a string of methods, which are available in one software package. The pipelines can distinguish between categorical covariates, i.e. for group comparisons or continuous covariates, i.e. for linear regression. The differential methylation analysis is often done internally by a linear regression without checking its inherent assumptions. A spike in the continuous covariate is ignored and can cause biased results.

**Results:**

We have reanalysed five data sets, four freely available from ArrayExpress, including methylation data and smoking habits reported by smoking pack years. Therefore, we generated an algorithm to check for the occurrences of suspicious interactions between the values associated with the spike position and the non-spike positions of the covariate. Our algorithm helps to decide if a suspicious interaction can be found and further investigations should be carried out. This is mostly important, because the information on the differentially methylated sites will be used for post-hoc analyses like pathway analyses.

**Conclusions:**

We help to check for the validation of the linear regression assumptions in a methylation analysis pipeline. These assumptions should also be considered for machine learning approaches. In addition, we are able to detect outliers in the continuous covariate. Therefore, more statistical robust results should be produced in methylation analysis using our algorithm as a preprocessing step.

## Background

Scientists using a linear regression model often ignore the properties of the independent variable or covariate. Especially, if the scientist is not aware of the use of a linear regression in differential expression analysis, because the regression analysis is hidden in the depths of a bioinformatical pipeline [1]. A classical at first glance unsuspicious continuous covariate might be smoking in pack years. A pack year is defined by the number of cigarette packs smoked per day multiplied by the number of years the person has smoked. One cigarette pack corresponds to 20 cigarettes. In a methylation study in the context of a differential expression analysis, we therefore want to model the change in methylation of a given CpG site by the amount of smoking pack years (SPY). In simple, the modeling is done by correlating the methylation values at the respective site with the smoking quantity for each patient and calculating a regression coefficient for smoking-dependent changes in methylation values. A problem in the fitting process occurs, if many non-smokers are included in the data analysis. Instead of a cloud of points, a single spike emerges at the left space of the covariate distribution. Other scenarios exist where a spike at the right is occurring. As a result, the regression line might be biased towards the spike patients (Additional file 1: Figure S1).

The modeling of an increased amount of zeros in the non-negative dependent variable *Y* is discussed extensively in statistic literature. If the outcome *Y* has a high amount of zeros, the outcome distribution is skewed to the left and must be modeled with care. This is called zero inflation. Different authors have proposed different solutions [2–7]. In contrast to these studies, we will concentrate on a spike at the left or right of the continuous covariate space. Hence, the covariate *x* is of interest and inflated with zeros. The covariate *x* has a spike of values at the left or at the right indicating the left or right limit of values, respectively. In principle, the proposed idea can also be used for categorical data, given a linear regression is appropriate for the analysis, i.e. an appropriate number of categories is available. To be concise, we are not looking at zero inflated data of the outcome, but at spikes at the limits of the covariate of interest. A possible example would be smoking pack years with a spike at zero for “non-smokers" and a continuous trend for “smokers" with different amounts of smoking pack years. To group continuous data into categories, it is common to define boundaries and then set all exceeding values to the corresponding limit. For example, the last group is often defined as “larger than” and clumping can occur. Sauerbrei et al. (2019) [8] stated, that the spike at zero modeling is relevant to the analysis of many studies, but practical experience is limited.

In the case of one outcome in a clinical study and a variable with a spike at zero, different approaches have been suggested to model the dependencies, demonstrated on data dealing with alcohol consumption as covariate and a single outcome to determine odds ratio effects in a dose-response setting [9, 10]. The modeling is always done on a few models with one outcome, like a single expression of a protein and different covariates, but not in the case of thousands of biomarkers like CpG sites. The experiment by Royston et al. (2010) [10] was designed to determine the relationship between the covariate, dosage, and one response. The authors conclude that if a spike at zero for alcohol consumption can be observed, fractional polynomials can be used for the modeling. Further, the approach was also extended into a setting with more variables with a spike at zero in the same model using a bivariate approach. In short, the spike part and the linear part are modeled by specific dummy variables indicating if an observation is included in the spike or not. However, software solution is not available in common bioinformatics languages like R or Python [11]. These type of modeling have also been used in the analysis of survey data in satisfaction with health care [7]. Lorenz et al. (2019) [6] applied their recent research on survival data and used the approach on a single protein expression in triple negative breast cancer [12]. Although the application has a bioinformatical background, the modeling was limited to one single protein and it was known, due to visual inspection, that a spike at zero was present. In contrast, in our work, we check thousands of biomarkers for the presence of a suspicious effect of the spike at zero. Lastly, Lorenz et al. (2017) [13] give examples and practical recommendations for the modeling of spike at zero. Lorenz et al. (2017) [13] summarize the actual state of the modeling of spike at zero including categorization of the covariate, fractional polynomial modeling, or the inclusion of a binary indicator of spike observations. They concluded after demonstrating on different biological examples, that general recommendations are difficult to provide. The analysis pattern depends on the main goal of the analysis. In our experimental case, we have thousands of endpoints that have to be checked.

A spike at the limits of the data space can be modeled. Especially at zero different methods are proposed by Jenkner et al. (2016) [11] and Lorenz et al. (2017) [13]. Both present approaches for low dimensional settings with one outcome and a set of covariates with a single spike at zero. In a genome-wide context thousands of biomarkers have to be modeled and only a fraction, if any, will have a spike at zero conflicting with the continuous covariate space. Therefore, biomarkers with a spike effect must be detected beforehand. Moreover, we must decide if we want to model the spike. From a mathematical point of view modeling can provide a better model explaining the variance by an improved fit. However, this might not match the biological point of view of the data modeling.

The bioinformatical analysis of high dimensional omics data is run in a pipeline fashion [14, 15]. This is feasible for the preprocessing and quality control of the samples until the differential analysis step begins [16]. In the case of an epigenome-wide association study (EWAS), not one CpG site is analyzed but hundreds of thousands. To model all the biomarkers with the assumptions of a spike as a part of the analysis pipeline does not make sense. First, a modeling including a spike will have more parameters and therefore will cost degrees of freedom resulting in statistically significant results being less likely. Second, to model data without a need does not fit the idea of a sparse model. If a spike has no influence on the data, the spike can be ignored and the continuous variable can be dichotomized. Therefore, the data can be analyzed by a simple group comparison with one group including the spike patients and the other group including the other patients. This can be done as long as no trend over the covariate can be observed. If a trend can be detected, the biomarker should be modeled differently. Nevertheless, a model including the spike is biologically misleading. If the spike supports the trend of the covariate, a simple linear regression can be conducted. If instead the spike is averaging out the effect or flips the direction of the effect, a severe biological interaction might be observable. In this case, a deeper look into the biomarker and its dependencies is needed and a simple modeling of the spike cannot be recommended.

In the following, we present an algorithm to detect suspicious interactions between values associated with the spike in the covariate and the non-spike associated values with the use of linear regression. We tested the algorithm on five methylation array data sets and checked if our proposed interactions are detectable in real life data or if the presence of a spike at zero is only a theoretical problem. Afterwards, we visualize the most suspicious interactions for each data set and show the arising problems. Overall, only a small margin of CpG sites show suspicious interactions between the spike and the linear part. However, in the analysis of EWAS, thoroughly scrutinized data sets are key to the subsequent detection of valid associations. Standard pipelines therefore include the filtering out of CpGs that lead to erroneous results, e.g., CpGs near SNPs (single nucleotide polymorphism) or cross-reactive probes. With this work, we present a method to overcome the problem of possible interactions between a spike and non-spike part introduced by a covariate and suggest its implementation into standard QC workflows for EWAS with continuous variables, therefore adding to the generation of reproducible results.

## Results

We ran the algorithms for spike at left (Algorithm 2) or spike at right (algorithm in Additional file 1 section 3) on all five data sets. No severe interactions were detected. Nevertheless, we were able to show effects of the corresponding spike and the reversal or negation of the linear effect by the spike in some CpG sites. Table 1 shows the results of the detection algorithm, the last two rows pointing towards the most important findings, indicating a reverse or negation influence of the spike.

**Table 1:** Results table of the ArrayExpress data and the data from Richter et al. (2019) [17]

Trend	E-GEOD				Richter ^*†*^
	32,861	54,643	55,454	68,825	
Negative linear trend	486	9,297	333	3,763	18,452
No linear trend	24,990	468,768	25,172	440,539	768,951
Positive linear trend	824	4,872	788	4,722	14,845
Reverse negative or negation	0	1,025	75	13	1
Reverse positive or negation	0	1,550	29	5	22
^*†*^ Richter et al. (2019) [17]					

The trend columns summarizes Figure 5 into five categories

In E-GEOD-32861, the spike showed no reverse or negation effects. 486 CpG sites showed a negative linear trend and 824 CpG sites have positive linear dependency with SPY. For the remaining 24,990 CpG sites, a normal group comparison between smokers and non-smokers would be feasible. Note that there was a gap between the SPY values of the non-smokers and the smokers. This might be a possible cause for no interaction between the spike and the linear part.

**Fig. 1:**
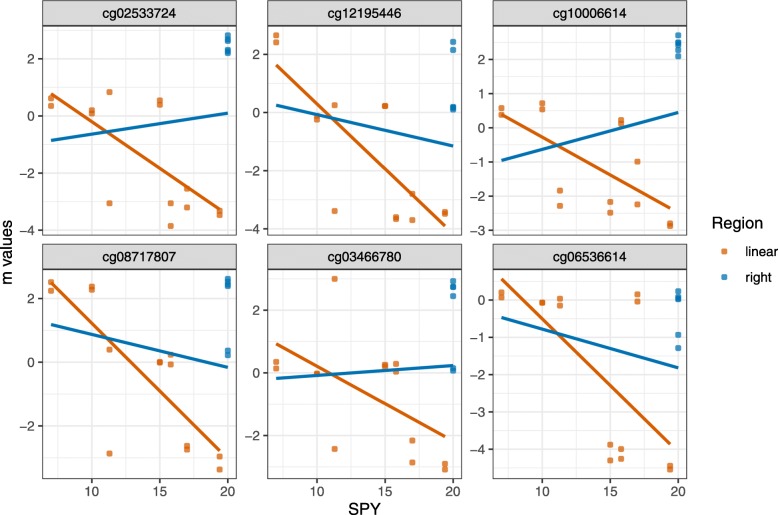
Top 6 of the reverse negative or negation CpG sites of the data set E-GEOD-54643. The order was determined by the effect deviation between the coefficients from two linear regressions, one associated with all data included and one with exclusion of the spike associated data. The spike at right increases the regression line. Without the spike the regression effect would be negative, including the spike at right the regression effect becomes positive. A biologist has to decide, if the CpG sites are biological connected or can be seen as outlier. Table 2 shows the position and genes nearby of the CpG sites

The E-GEOD-54643 data showed 1,025 reverse negative or negation and 1,550 reverse positive or negation CpG sites. Further, 9,297 CpG sites showed a negative linear trend and 4,872 have a positive linear dependency. The majority of the CpG sites, 468,768, can be analyzed by a group comparison between smokers and heavy smokers. Table 2 shows the Top 6 of the reverse positive or negation CpG sites pictured in Fig. 1. The results must be judged carefully because of the low sample size. Nevertheless, we could demonstrate our concerns of a linear regression on covariates with a spike at a given position on this example. Most of the Top 6 findings were CpG sites mapped in genes with clinically relevant functions. The methylation site cg12195446 interacts with genes controlling the insulin household, cg10006614 is included in the epithelial cell morphology, cg03466780 negatively regulates the elongation of transcription by RNA polymerase II and cg06536614 is near the gene TGFB1, which codes an important growth factor.

**Table 2:** Genetic summary table of the results of the Top 6 findings in E-GEOD-54643

CpG ID	Chr	Start	End	Gene ID	
				included	nearby
cg02533724	10	128,481,648	128,481,697		LINC01163 (ncRNA); AL390763.1 (ncRNA)
cg12195446	13	109,772,102	109,772,151	IRS2	
cg10006614	14	95,410,951	95,411,000	SYNE3	
cg08717807	16	85,497,808	85,497,857	GSE1	
cg03466780	9	137,352,913	137,352,962	NELFB	
cg06536614	5	136,080,692	136,080,741		TGFB1

Figure 1 shows a strong deviation between the inclusion and the exclusion of the spike at 20 SPY of the linear regression

**Fig. 2:**
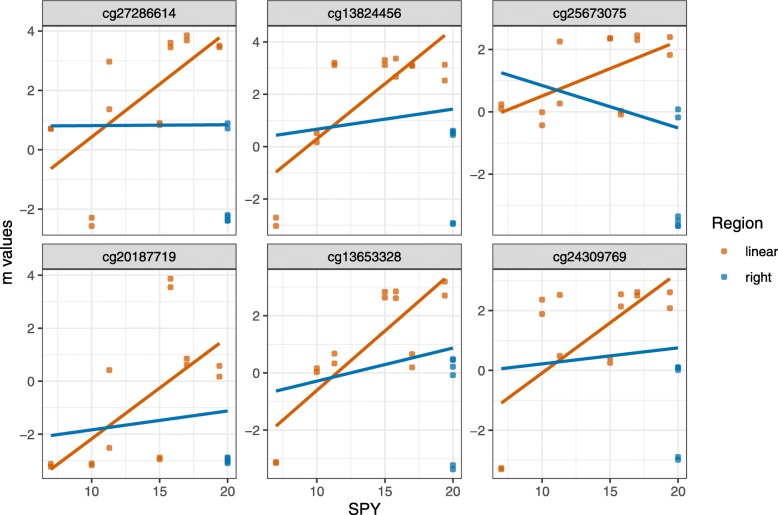
Top 6 of the reverse negative or negation CpG sites of the data set E-GEOD-55454. The order was determined by the effect deviation between the coefficients from two linear regressions, one associated with all data included and one with exclusion of the spike associated data. In contrast to E-GEOD-54643 the spike at right decreases the regression line. Without the spike the regression effect would be positive, including the spike at right the regression effect becomes negative. Again, a biologist has to decide, if the CpG sites are biological connected or can be seen as outlier. Table 3 shows the position and genes nearby of the CpG sites

E-GEOD-55454 had 75 RNN and 29 RPN CpG sites. Figure 2 shows the results of the Top 6 strongest deviations between the two regression lines. The CpG site cg00073650 showed a strong effect through two outliers in the lower region of the SPY values. Due to the outlier, the regression line had a higher slope and the predicted value for the spike at right at 60 SPY was higher than the threshold. The methylation site cg00231920 is located in the genes TMEM23 and PCED1A, which both play a role in the generation of transmembrane proteins. CpG site cg01352108 maps to KCKNK4, which is connected to the perception of pain caused by heat, cg02699167 is utilized for formation of membranes, cg05421673 plays a role in the inhibition of bone morphogenetic proteins, and cg06220521 has an important role in the regulation of vascular remodeling.

**Table 3:** Genetic summary table of the results of the findings in E-GEOD-55454

CpG ID	Chr	Start	End	Gene ID	
				included	nearby
cg00231920	20	2,840,614	2,840,663	TMEM239; PCED1A	
cg01352108	11	64,291,358	64,291,407	KCNK4	
cg02699167	3	33,277,532	33,277,581	FBXL2	
cg05421673	17	56,594,214	56,594,263	NOG	
cg06220521	13	94,601,914	94,601,963	GPR180	

Figure 2 shows a strong deviation between the inclusion and the exclusion of the spike at 60 SPY of the linear regression

E-GEOD-68825 had 13 RNN and 5 RPN CpG sites. Additional file 1: Figure S6 shows the Top 6 of CpG sites with the largest differences between the two regressions models. Demonstrating the negative effect of the inclusion of the spike into the regression model, Additional file 1: Figure S7 shows again strong effects from possible outliers. As example, the CpG sites cg09270247 and cg25457956 showed a strong positive trend, which was negated by the inclusion of the spike at 50. It seems that the methylation was increasing until 50 SPY and was dropping to a more constant level. Scientists should investigate these samples with care and decide, whether they should be excluded or whether they could include more insight into unseen complex biological backgrounds. The effects were not very large and in addition with the lack of information on the data set, we will not go deeper into the results.

**Fig. 3:**
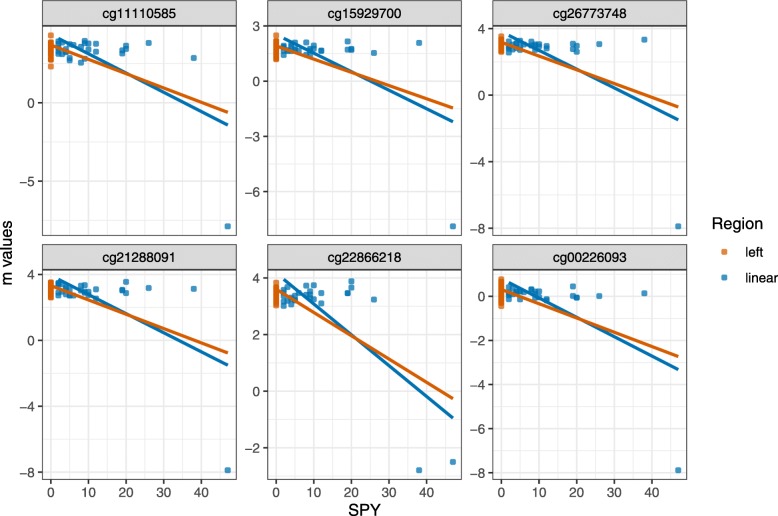
Top 6 of the reverse positive or negation CpG sites of the data set by Richter et al. (2019) [17]. The order was determined by the effect deviation between the coefficients from two linear regressions, one associated with all data included and one with exclusion of the spike associated data. In contrast to E-GEOD-54643 and E-GEOD-55454, the spike effect is very marginal. Both regression lines have nearly the same slope with the same negative effect. It is clear to see that an outlier is causing the suspicious interactions

The data by Richter et al. (2019) [17] showed 1 RNN and 22 RPN CpG sites. Figure 3 shows the Top 6 of the strongest deviations of both regression analyses for the reverse positive or negation CpG’s. All effects were driven by one outlier, which had not been not included in the original analysis by Richter et al. (2019) [17]. The outlier with a SPY value of 47 had very low *m*-values. Removing this patient from the analysis would reduce the number of suspicious interactions to zero.

In summary, we were able to show the effect of the inclusion of the spike and the non-spike values in a linear regression. In the case of a low sample size the spike has a larger effect than in the case of a higher sample size. We were able to show, that outliers in the non-spike values might also influence the regression analysis. These outliers might not be detected by preprocessing because the outliers are directly connected to the covariate. Checking the validity of the assumptions is crucial for the biological interpretation of the statistical analysis. The results of the linear regression in our algorithm can only be seen as a preprocessing step to detect suspicious CpG sites connected with SPY. The researcher should remove the detected CpG sites and discuss these CpG sites separately. Our algorithm does not guide any statistical decisions for the differential analysis for the other CpG sites.

## Discussion

Why do we not model the spike in the covariate distribution? In bioinformatics, the analysis of a high amount of biomarkers like CpG sites in epigenome-wide association studies is common. In our work, we present a way to address this challenge, i.e. detect suspicious interactions between the spike values and the other values of the covariate before streamlined association analyses. The general idea would be to model these dependencies. As only a small fraction of all CpG sites will be influenced by the spike, we do not consider this approach appropriate. If we would model all the biomarker considering the spike and use more complex models like fractional polynomials, we would face the following problems: First, the model will often be more complex than needed. We would need to estimate more shape parameters for the fractional polynomials. This would violate the sparsity rule of a good model and will cause a lower power because of the usage of more degrees of freedom. Hence, less significant results can be found. More importantly, for the vast majority of biomarkers, the model will be more complex than needed. The linear regression model is a simple and well suited model and should be used if the assumptions are valid. We present a possibility to detect CpG sites, which are violating the assumptions and must be verified visually. Guided by the results of our algorithm, it can be evaluated whether the suspicious interactions are statistical noise or whether the CpG sites may have an interesting biological context that would explain unexpected spike effects. This cannot and should not be done simply by statistical modeling.

One could however argue that we introduce a pretest to a bioinformatical analysis pipeline. This is partly true. However, to avoid multiple testing problems and to avoid an increase of the type I error due to many significance test, we recommend to look and check only the suspicious CpG sites. If the CpG site is in line considering the spike and the non-spike data, a post-hoc analysis as planned can be conducted. In this post hoc linear regression all batch effects and other confounders can be included and adjusted for. Therefore, our tested model is very simplistic and has the only aim to check for the spike effects. We can not recommend to use any effect measures from this analyses for the biological interpretation. Bourgon et al. (2010) [18] shows in his work the importance of independent filtering and the connected increase of detection power for high-throughput experiments. Hence, it is important that the researcher decides, if possible detected suspicious interaction will violate the linearity assumption of the regression analysis or if the independent assumption of the bioinformatical pipeline can not hold. We would state, that a flipped or misleading effect is much more problematic than a lower statistical power [1, 16].

As a side effect, our detection algorithm also allows to run a quality check for outliers considering the covariate with a spike, which would bias the differential association analysis. The algorithm supports the decision on whether a variable should be dichotomized or not. If no linear trend can be observed in the non-spike associated data values, it might be feasible to run a simple group comparison or a means parametrization using a linear regression with confounder adjustment. Again, we strongly recommend to use a more complex model with confounder adjustment for the full statistical analysis followed by biological interpretation of the estimates.

If the experiment includes a high number of samples, we would strongly recommend to change the decision rule for the detection of a linear effect in the non-spike values. With an increase of the sample size, we will observe significant linear results even if the clinical effect is small and therefore ignorable. Hence, we have added the possibility to define a clinical threshold, which must be reached to have a linear dependency in the non-spike data. This must be decided manually by the scientists, who runs the spike detection algorithm. Taking our findings into account, only a small fraction of biomarkers should be suspicious. If a large number of suspicious biomarkers is observed, the cause might be the high sample size.

Differential analysis is important for further analysis steps in a bioinformatical analysis pipeline. Significant biomarkers are processed further on in pathway or enrichment analysis. From a statistical point of view the analysis of the data should hold the 5% family wise error rate. At most 5% of the biomarkers should be significant although the null hypothesis is true and no real effect of the methylation or expression is present. The false discovery rate (FDR) allows to choose a more liberal approach and to utilize the full significance level. To achieve significant results in genetics is very important. After the differential analysis a gene set enrichment or pathway analysis is often conducted using the significant results of the differential analysis. Hence, if only a low number of biomarkers is significant, the enrichment analysis has problems to detect differentially expressed pathways. As mentioned above the independence of the filtering is important [18]. Nevertheless, Allen (2017) [19] describes the problems with multi omics data integration if not each omics layer does not include outlier and is well preprocessed. If the different layers should be combined, each of them should be conform as expected. Spikes in the covariate are not typical in statistical modelling and problematic interactions in biomarker should be removed beforehand.

From our point of view, not the low power is a problem, though it is possible that one does not detect a potential true pathway. However, a more severe problem would be the direction of the effect. If the spiking covariate flips the effect measure of the linear regression, the whole pathway might be directing in the wrong direction. A potential protective pathway will become a potential risk pathway or vise versa. If all CpG sites are more or less in a linkage disequilibrium, then one suspicious effect of the covariate should be found in all CpG sites connected to a given pathway. How strong this suspicious effects must be is open for further research.

Further, in epigenetics many new approaches for machine learning are proposed and used in direct application [20, 21]. We will need data for machine learning which hold the assumptions to the data. If not, we will not model the dependencies in the data space but most likely the spike effect, which will be very dominant. Especially, if we want to use machine learning on the data, we must know if interactions are present. In simple machine learning, approaches try to model the correlation structures. A spike at a given position, which is not in consensus with the rest of the data might cause a bias in the prediction algorithm.

## Conclusion

We demonstrated in our work an algorithm to detect interactions between the spike at the left or the right of a continuous covariate in the setting of a differential expression analysis. If the spike of the covariate is not in conjunction with the linear part of the other values of the covariate, a linear regression could deliver biased estimates. Differential analysis based on significant CpG sites and maybe swapped estimated effects of the linear regression slope will be misleading. Our proposed algorithm can be used for each covariate with a known spike after preprocessing. Suspicious biomarkers can then be checked visually. The scientist can decide, if the respective biomarker should be included for further analysis or dismissed. In rare cases such a biomarker with a suspicious interaction might be of special interest, especially if many of the genes are located in the same genetic region. Further, our approach can also be used for the detection of potential outliers, which would bias the linear regression. Finally, we used the algorithm on five real life data sets and detected only slight deviations, mainly driven by low sample sizes. Still, we would advice to check the covariate, if the genetic study includes potential spiking covariates. Machine learning, enrichment analysis or pathway networks are often based on differentially expressed findings and will be more robust, if the assumptions on the differential analysis were valid.

## Methods

### The biological model

In this work, we concentrate on the distribution of the covariate in a linear regression model. A genetic data set consists of thousands of biomarkers, which are all analyzed in the same way in a pipeline fashion. In the context of this work, we assume the investigated biomarkers to be CpG sites in an EWAS. In a simple setting of a covariate representing two treatment groups, the analysis is straightforward. However, sometimes the covariate of interest is not binary like smoking but continuous, like smoking pack years (SPY) has many groups with order, like the ASA physical status classification system, or has joystick years as a analogy to smoking pack years. In the study of Kuehn et al. (2014) [22] the influence of joystick years on different regions of interest (ROI) in the brain was analysed. With an increased number of considered voxels, i.e. ROI’s, the problem of undetected spikes at zero could occur.

In this work, we will concentrate on the differential analysis of CpG sites in EWAS. For the analysis of EWAS, two large R packages are available: Champ [23] and minfi [24]. Nevertheless, in both cases the differential analysis is based on the limma package [25]. Therefore, the differential analysis is done by a linear regression adapted for the outcome with a linkage function or by adjusting the variance with a Bayesian approach [26]. Subsequently, the regression analysis can be followed by a gene set enrichment analysis or pathway analysis, depending on the statistical outcome. In a normal setting the assumptions on the covariate are neglected. In the special case of smoking pack years a spike at zero can be observed in the covariate distribution. Has this spike an effect on the estimates of the regression analysis?

### Algorithm for the detection of suspicious interactions

In the following, we present an algorithm to detect suspicious interactions between methylation values of the patients included in the spike and the methylation values associated with the non-spike values of the covariate of interest. So called *β*- or *m*-values usually represent methylation values. A *β*-value can be roughly defined as the percentage of methylation in one CpG site. When statistical analysis is performed, *m*-values, which are based on *β*-values, are used to gain a higher statistical accuracy. The *m*-values are retrieved by dividing the methylated fraction (*β*-value) of a CpG site by the unmethylated fraction (1−*β*-value) and then taking the natural logarithm of 2 of this outcome. This leads to a possible range of −*∞* to *∞* for the *m*-values. In the following, *m*-values represent methylation values. In addition, we decided to use smoking pack years as the spiking covariate as an example. In general, every variable with “non-users” and “users” can have the property of a spike. Further, we also introduce the detection of a spike on the right side of the value space like a censoring of measurement values or a wanted maximum value. In the case of smoking, we could think of a group of heavy smoker, which spike on the right at a given SPY value.

**Fig. 4:**
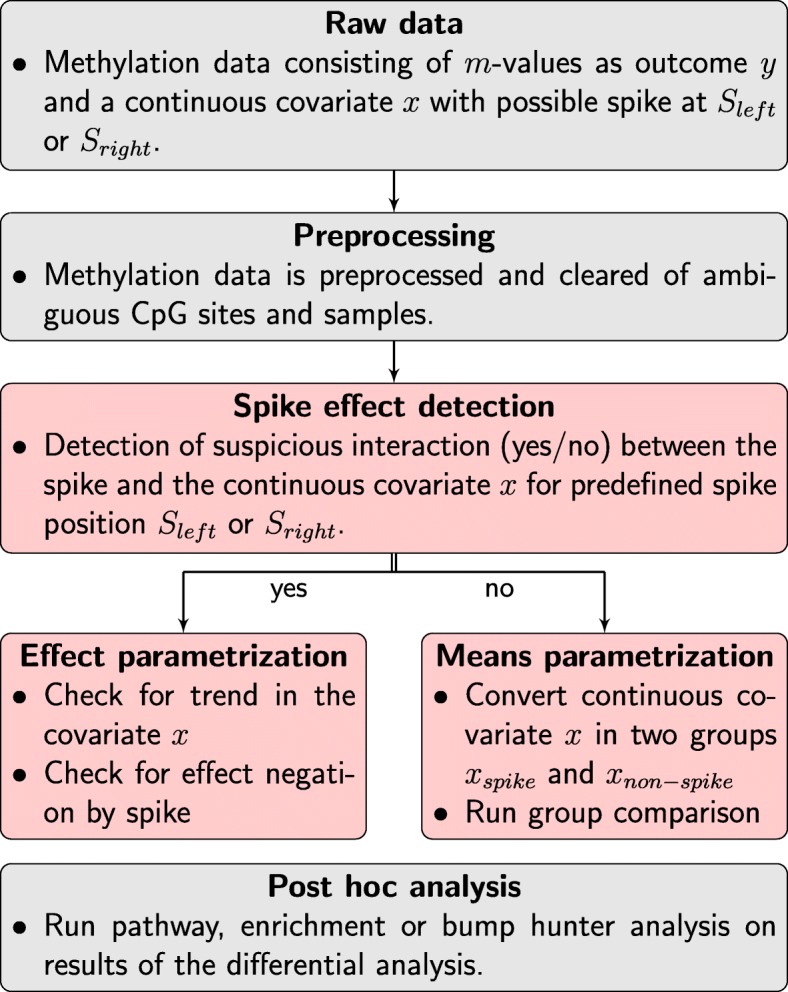
Flowchart of integration of the detection of suspicious interactions in the general workflow of a methylation analysis pipeline. After the preprocessing, the spike effect detection is run to determine the CpG sites which must be inspected visually if a relevant deviation is present. Afterwards, normal post-hoc analysis can be run on the data

Figure 4 shows the inclusion of our spike effect detection algorithm in the general frame work of a methylation analysis. First, standard preprocessing methods, available in the common bioinformatics pipelines, will be used for preprocessing raw data consisting of *m*-values and a continuous covariate *x* with a known spike position. In the context of this work, we will not include any batch effects or other confounders, but will only check, whether the spike validates the linear dependency of the *m*-values or has an altering interaction with non-spike values. After the preprocessing of the *m*-values, the covariate *x* with a known spike position will be checked for introduction of suspicious interactions between *m*-values associated with the spike and a possible linear effect of the non-spike associated *m*-values. Afterwards, a normal differential analysis can be run. The association of the *m*-values with the non-spike part of the covariate *x* will in the following also be referred to as the linear part of the covariate *x* and an effect of this linear part will be called linear effect of the covariate *x*.

**Fig. 5:**
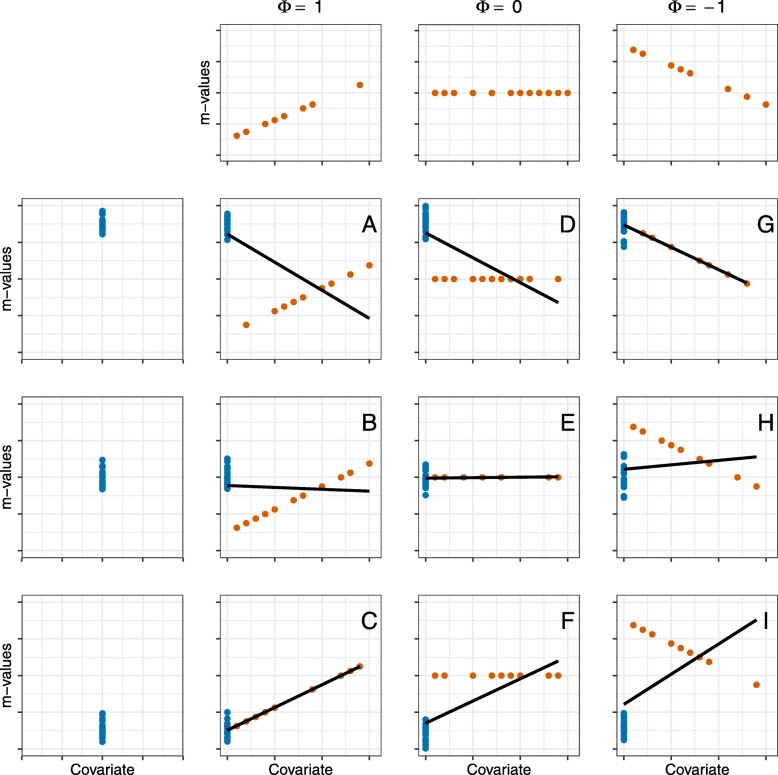
Demonstration of possible interactions between a spike at zero and the trend in the remaining values of the covariate on an example of normal distributed *m*-values generated by an Epigenome-wide association study. The covariate could have different spike at left positions shown in the left columns: high, medium, and low values. The remaining, non-spike covariate values could have a positive trend (A, B, and C), no trend (D, E, and F) or a negative trend (G, H, and I). The position of the spike and the trend can support each other (C, E, and G), cancel each other out (B and H), or switch the biological interpretation (A and I)

Figure 5 shows the possible outcomes of our spike effect detection algorithm as a 3x3 plot matrix illustrating the relationship between *m*-values associated with the spike values and the linear effect of the non-spike associated *m*-values. The left column shows the position of the spike. Although our algorithm is also able to detect a suspicious interaction with a spike at the right of the covariate values, in this Fig. 5, we concentrate on the spike at the left (see Additional file 1 section 3 for the algorithm of the detection of the spike effects at the right). The spike can include low, mid or high values, as indicated by the fourth, third and second row, respectively. In the first row, the linear effect of the covariate is shown. The effect can be increasing, stable with no effect or decreasing. We combine these three states of the spike, low, mid and high, with each of the three possible tendencies of the covariate, increasing, stable and decreasing.

First, the spike position can be in line with the linear effect of the covariate, as depicted in subplot C and G. The spike is located lower than the linear part and the linear effect is increasing (Fig. 5C) or the spike is located higher than the linear part and the linear effect of the covariate is decreasing (Fig. 5G). Hence, the spike and the linear effect of the covariate are in the same direction and a normal linear regression is feasible. Second, if we observe no linear trend among the covariate, as shown in subplot D, E, and F, a normal group comparison between the spike and non-spike individuals is possible. We suggest to use a linear regression with means parametrization to compare both groups and adjust for further confounders if needed. Third, suspicious interaction occurs if the spike will negate or reverse the effect observed in the linear part of the covariate, as in settings A and B or H and I. This will not occur often, but if we observe such a pattern, we must investigate these CpG sites first before we can go on with the analysis pipeline.

How does the algorithm decide which of the scenarios depicted in Fig. 5 applies to the *m*- and SPY values in a given CpG site? First, we need to determine whether a linear (clinical) effect *ϕ* can be observed in the *m*-values associated with the non-spike part of the covariate. Next, if a linear effect *ϕ* can be observed, we must decide in which direction the effect is pointing. It would be the easiest if *ϕ* was determined by the clinical investigator beforehand. Often this decision is not possible, because the information on the relevant clinical effect *ϕ* with emphasis on the outcome *m*-values is not known. Algorithm 1 shows the determination of the linear (clinical) effect *ϕ* of the covariate. It sets *ϕ* based on the *p*-value of the *β*-coefficient associated with the non-spike part of the covariate and the sign of this *β*. For *ϕ*, the values 1, −1 or 0 are returned by Algorithm 1 representing a positive effect, negative effect or no effect, respectively. Hence, we will say that there is a clinical relevant effect, if a significant trend can be observed. We use the sign of the regression coefficient to decide if the trend is increasing or decreasing. If the covariate shows a increasing trend, column two (*ϕ* = 1) of Fig. 5 is possible. One of the interactions depicted in column four (*ϕ* = −1) is feasible if the covariate shows a decreasing trend. Lastly, if *ϕ* is 0, column three is achievable.


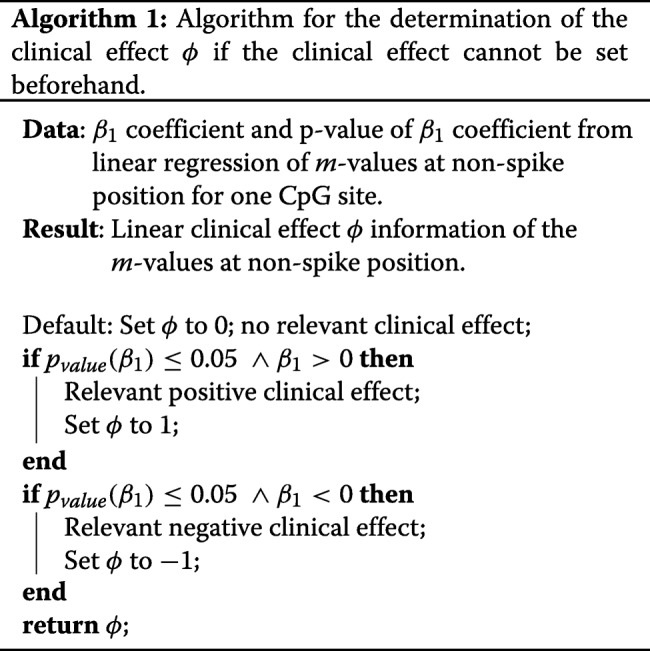


After we have decided whether a clinical effect can be observed, we can look at the interaction between the spike and the linear part. Again, if no clinical linear effect can be observed, the given CpG site will not be investigated further for suspicious interactions. Algorithm 2 shows the whole sorting and effect detection algorithm for the spike at left. The algorithm runs for all CpG sites. We define the spike position on the left, normally zero. First, we run a linear regression without the spike data. Second, as part of Algorithm 2, we determine the clinical effect *ϕ* with Algorithm 1. If a trend can be observed in the linear part of the covariate, we must decide if a suspicious interaction between the spike can be found. Is the spike in conjunction with the direction of the linear regression? Hence, we set the mean of the spike associated *m*-values minus two times the standard deviation as *Q*_1_ and the mean plus two times the standard deviation as *Q*_3_. If we observe a positive linear trend and the *β*_0_ coefficient of the linear regression is lower than *Q*_1_, a suspicious interaction can be observed, indicated by subplot A and B in Fig. 5. If a negative trend of the covariate can be observed and the *β*_0_ coefficient of the linear regression is greater than *Q*_3_, again, a suspicious interaction can be observed. Then, we are located in the subplots H and I of Fig. 5.


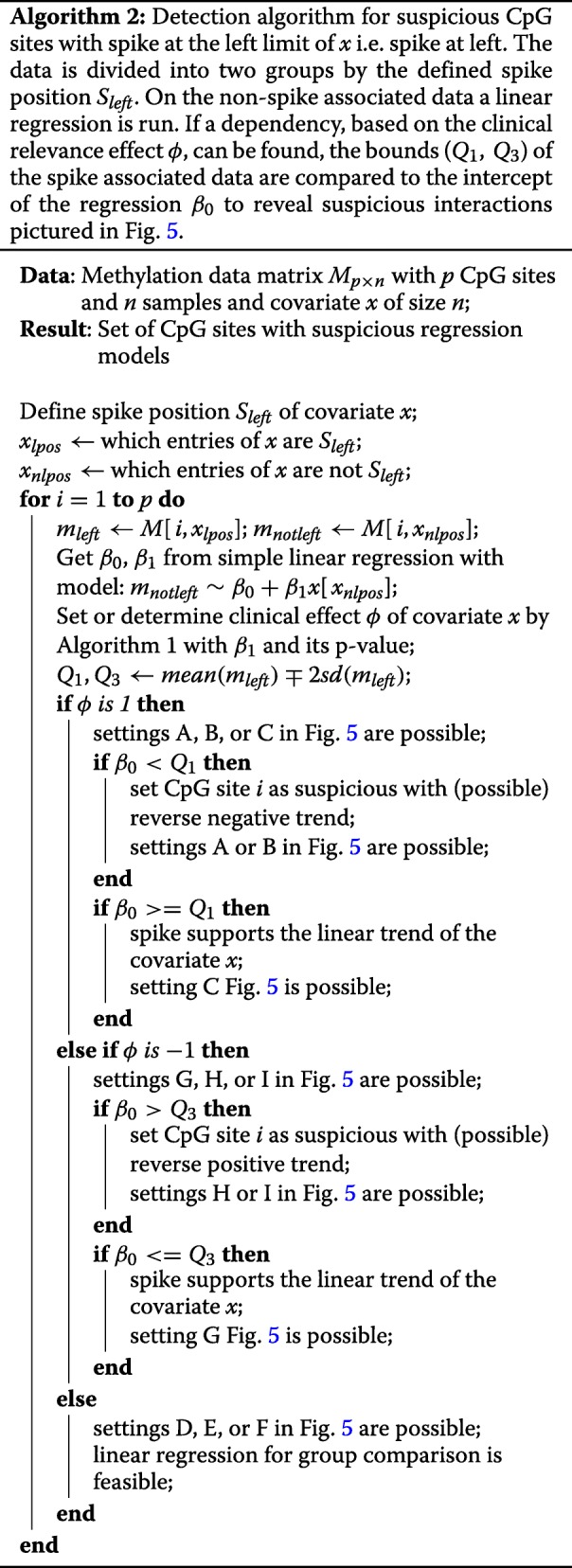


Because we can not really distinguish between subplots A and B as well as H and I, we collapse them to “Reverse negative or negation (RNN)” and “Reverse positive and negation (RPN)”, respectively. The subplots D, E, and F will be named “No linear trend”. The subplots C and G will be called “Positive linear trend (PLT)” and “Negative linear trend (NLT)”, respectively.

If we would assume a spike at the right, a swap in the decision rules occurs. Further, we will not only look at the *β*_0_ coefficient of the linear regression but the predicted value by the linear regression at the spike position. This predicted value will then be compared to *Q*_1_ and *Q*_3_. The algorithm for the spike at right detection can be found in the Additional file 1 section 3. Most importantly, the user must define the spike position at the right before the use of the Algorithm 2.

We used the algorithm to detect suspicious interactions in five data sets, of which four are publicly available, to check the appearance of such interactions. Overall only a few data sets included an observable effect. Nevertheless, the interaction can cause problems later in the pipeline and should therefore be checked and considered.

For ranking of the CpG sites with suspicious interactions, we ran two linear regressions on each CpG site. Again, the *m*-values were the response and the SPY values served as the covariate *x*. One linear regression model included both spike and non-spike patients and the other excluded spike patients. The ranking was then based on the deviation of the two linear regression coefficients associated with the covariate *x*. The presented algorithm is available as R code in the Additional file 2.

### Data sets for the spike effect detection

We searched the ArrayExpress data base for data sets including methylation profiling and smoking habits by smoking pack years. Hence, we used the search term <“Methylation profiling by array” & “pack years” > to find overall six experiments. We downloaded the processed files and the phenotype data of the experiments with the following accession numbers: E-GEOD-32861, E-GEOD-32867, E-GEOD-54643, E-GEOD-54690, E-GEOD-55454, E-GEOD-68825. The experiments E-GEOD-32861 and E-GEOD-32867 are associated with the same source. Therefore, we decided to reanalyze the experiment with the larger available sample size, namely experiment E-GEOD-32861. Furthermore, E-GEOD-54690 had the same number of patients with the same entries of SPY as E-GEOD-54643. We decided to analyze E-GEOD-54643 as the data is the same. In addition, we were able to reanalyze the data from Richter et al. (2019) [17] with a larger sample size than analyzed in the publication. This was possible as we ignored assessment problems and lab quality of all samples. Finally, five data sets were analyzed for the existence of spikes in the covariate smoking pack years (SPY). Table 4 shows a summary of the ArrayExpress data and Richter et al. (2019) [17] data. In the following, we will describe the analyzed data in more detail.

**Table 4:** Summary table of the ArrayExpress data and the data from Richter et al. (2019) [17]

	E-GEOD				Richter ^*†*^
	32,861	54,643	55,454	68,825	
Samples	118	20	38	113	75
CpG sites	26,300	485,512	26,397	449,042	802,271
SPY	25.31±32.48	17.15±6.57	49.85±24.84	51.54±31.46	4.33±8.62
	[0; 120]	[7; 27.7]	[13; 156]	[8; 192]	[0; 47]
Spike at ^*‡*^	0	20	60	50	0
Spike samples	62	6	7	49	45
Linear samples	56	14	31	64	30
^*†*^ Richter et al. (2019)[17], ^*‡*^ according to publication					

If the spike for SPY was not at zero, we set the spike accordingly

The E-GEOD-32861 data set was taken from the work of Selamat et al. (2012) [27]. The data consisted of 118 preprocessed samples and 26,300 CpG sites and had been acquired with the Illumina Infinium HumanMethylation27 BeadChip. We looked at SPY as the covariate with a possible spike at zero. The SPY values ranged from 0 to 120 with a mean of 25.31±32.48. Therefore, we set the spike at left to zero. We had 62 samples with a SPY of zero, possible “non-smokers”, and 56 samples with different values of SPY. However, the SPY zero values were misleading in this study. “Never-smokers” were defined as having smoked less than 100 cigarettes a lifetime. The SPY range of only the smokers was between 11 and 121. This must be kept in mind for the discussion of the results of this study by the spike effect detection.

Milenkovic et al. (2014) [28] had generated the E-GEOD-54643 data. The data consisted of a small sample size of only 20 individuals with 485,512 CpG sites, acquired with the Illumina Infinium HumanMethylation450 BeadChip. Only smokers were included in the study. The covariate SPY’s mean was 17.15±6.57 and it ranged from 7 to 27.7. Therefore, we looked at the histogram of the SPY values and decided for a spike at right at a SPY value of 20. Hence, 6 patients were grouped into the spike group and 14 into the linear part. Due to the small sample size, we expected more extreme outcomes.

Vucic et al. (2014) [29] had produced the data of E-GEOD-55454. The study consisted only of 38 former smokers and 26,397 CpG sites on an Illumina Infinium HumanMethylation27 BeadChip. We observed a mean SPY of 49.85±24.84 with a range from 13 to 156. After looking at the histograms of the SPY values we decided to generate a spike at the right at 60 SPY. Therefore, we determined 7 spike samples and 31 non-spike samples.

The accession number E-GEOD-68825 had no connected publication and had been titled “Analysis of DNA Methylation for LUSC using Illumina Infinium HumanMethylation450 platform”. The data consisted of 113 samples with 449,042 CpG sites, acquired with the Illumina Infinium HumanMethylation450 BeadChip. The SPY values ranged from 8 to 192 with a mean value of 51.54±31.46. We decided after consulting the histograms of the SPY value to set the spike at right at 50. Hence, we determined 49 spike patients and 64 non-spike samples. No further information on the data was available at ArrayExpress.

Finally, we reanalyzed Richter et al. (2019) [17] on a data basis larger than in the publication, to show possible complications. The data consisted of 75 samples and 802,271 CpG sites that had been run on an Illumina Infinium DNA MethylationEPIC BeadChip. In the publication, the authors had preprocessed the data to remove reactive probes and other ambiguous CpG sites resulting in 39 samples being analyzed. We used the larger set of available samples to demonstrate the algorithm for the spike effect detection. In this data set, we observed SPY values from 0 to 47 with a mean of 4.33±8.62. We set the spike position at the left with a spike at zero. Hence, we had 45 patients in the spike group and 30 in the non-spike group.

## Supplementary information

**Additional file 1** Supplementary material to the spike at right detection and further figures of the data with the identifiers E-GEOD-32861, E-GEOD-54643, E-GEOD-55454 and E-GEOD-68825 are available at ArrayExpress.

**Additional file 2** R code and example of the Algorithms 1 and 2 for the detection of suspicious spike interactions.

## Data Availability

The software is available in the supplementary material in an extra R file. The data with the identifiers E-GEOD-32861, E-GEOD-54643, E-GEOD-55454 and E-GEOD-68825 are available at ArrayExpress (https://www.ebi.ac.uk/arrayexpress/). Simply use the identifier as search term.

## Abbreviations

ASA physical status classification system: American Society of Anesthesiologists physical status classification system
CpG Site: position of a methylation
DNA: Deoxyribonucleic acid
EWAS: Epigenome-wide association study
NLT: Negative linear trend
PLT: Positive linear trend
QC: quality control
RNN: Reverse negative or negation
RPN: Reverse positive and negation
SNP: Single nucleotide polymorphism
SPY: Smoking pack years
