# Supplementary material for: Detection of suspicious interactions of spiking covariates in methylation data
Source: BMC Bioinformatics. 2020 Jan 30;21:36. doi: 10.1186/s12859-020-3364-6 (PMC6993406; doi:10.1186/s12859-020-3364-6)
Supplement: Supplementary file 1 — Additional file 1 Supplementary material to the spike at right detection and further figures of the data with the identifiers E-GEOD-32861, E-GEOD-54643, E-GEOD-55454 and E-GEOD-68825 are available at ArrayExpress. [file 12859_2020_3364_MOESM1_ESM.pdf]

Supplementary material

Detection of suspicious interactions of spiking covariates in methylation data

Miriam Sieg[1,2], Gesa Richter[3], Arne Schaefer[3], and Jochen Kruppa[1,2*]

[1]Charité - University Medicine, corporate member of Freie Universität Berlin, Humboldt-Universität zu Berlin, and Berlin Institute of Health, Institute of Biometry and Clinical Epidemiology
[2]Berlin Institute of Health (BIH), Berlin, Germany
[3]Department of Periodontology and Synoptic Dentistry, Institute of Dental, Oral and Maxillary Medicine, Charité - University Medicine
[*]corresponding author

Last changed: September 18, 2019

Contents

1 Overview of the supplementary material **3**

2 Theoretical data example **4**

3 Spike at right algorithm **5**

4 TOP 6 of suspicious interactions **6**

1 Overview of the supplementary material

In the following additional tables and figures of the paper is provided.

Supplementary tables No supplementary tables are delivered.

Supplementary figures Figure 1 shows the theoretical data setting of the covariate and the possible spike at the left or at the right. Remember, we can only model one spike position. Therefore, the data has a known spike position at the left or at the right.

For a better overall overview and comparison, we included all figures of suspicious interactions including figures shown in the connected publication.

Figure 2 and figure 3 show the negation of the positive and negative linear effect by the spike at right in the sample E-GEOD-54643.

Figure 4 and figure 5 show the negation of the positive and negative linear effect by the spike at right in the sample E-GEOD-55454.

Figure 6 and figure 7 show the negation of the positive and negative linear effect by the spike at right in the sample E-GEOD-68825.

Figure 8 and figure 9 show the negation of the negative and positive linear effect by the spike at left in the sample of Richter *et al.* (2019)[1].

Supplementary algorithms Algorithm 1 shows the detection of a spike at right. The main difference is the definition of β_o. We set β_0 to the predicted value by the linear regression for $x \not\subset S_{right}$ on the spike position. Technically, this is not β_0 from a linear regression. In addition, decision rules are swapped, if the spike is at the right. Therefore, the regression line is under the spike, if the regression line decreases and vice versa.

2 Theoretical data example

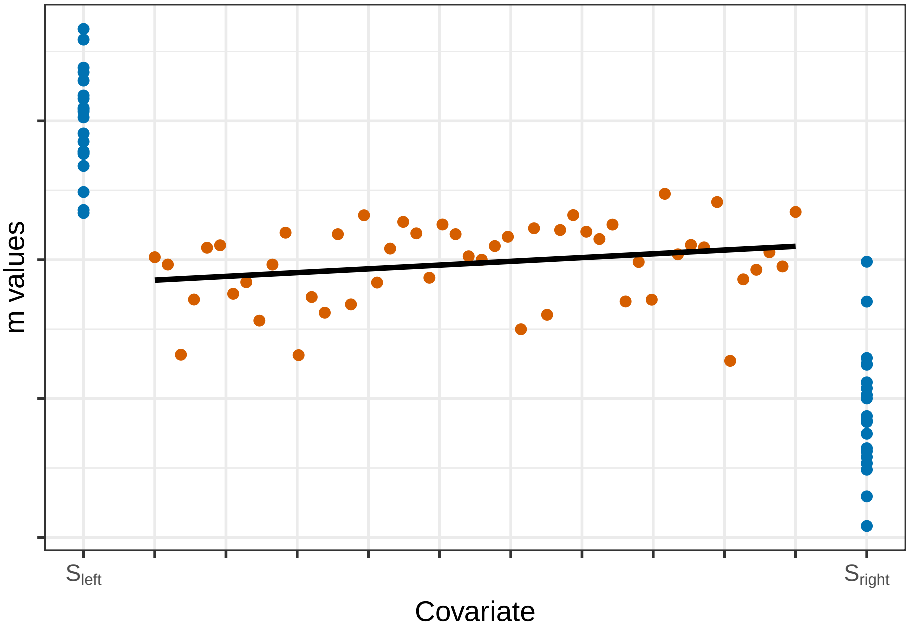

Supplementary Figure 1: Example data set of one CpG site with a spike at left and spike at right. A non binary outcome is dependent on a non negative covariate with a spike at the left or right of the variable space. A regression analysis is the aim of the followed analysis to reveal the dependency between the outcome and the covariate indicated by the black line.

3 Spike at right algorithm

Algorithm 1: Detection algorithm for suspicious CpG sites with spike at the right limit of x i.e. spike at right. The data is divided into two groups by the defined spike position S_{right}. On the non-spike associated data a linear regression is run. If a dependency, based on the clinical relevance effect ϕ, can be found, the bounds (Q_1, Q_3) of the spike associated data are compared to the predicted value of S_{right} through the regression model to reveal suspicious interactions.

Data: Methylation data matrix $\boldsymbol{M}_{p \times n}$ with p CpG sites and n samples and covariate x of size n;
Result: Set of CpG sites with suspicious regression models

```
Define spike position S_right of covariate x;
x_rpos ← which entries of x are S_right;
x_nrpos ← which entries of x are not S_right;
for i = 1 to p do
    m_right ← M[i, x_rpos]; m_notright ← M[i, x_nrpos];
    Get β0, β1 from simple linear regression with model: m_notright ~ β0 + β1 x[x_nrpos];
    Set or determine clinical effect φ of covariate x by algorithm 1 with β1 and its p-value;
    Q1, Q3 ← mean(m_right) ∓ 2sd(m_right);
    if φ is 1 then
        if (β0 + β1 * S_right) > Q3 then
            set CpG site i as suspicious with (possible) reverse negative trend;
        end
        if (β0 + β1 * S_right) <= Q3 then
            spike supports the linear trend of the covariate x
        end
    else if φ is −1 then
        if (β0 + β1 * S_right) < Q1 then
            set CpG site i as suspicious with (possible) reverse positive trend;
        end
        if (β0 + β1 * S_right) >= Q1 then
            spike supports the linear trend of the covariate x;
        end
    else
        linear regression for group comparison is feasible;
end
```

4 TOP 6 of suspicious interactions

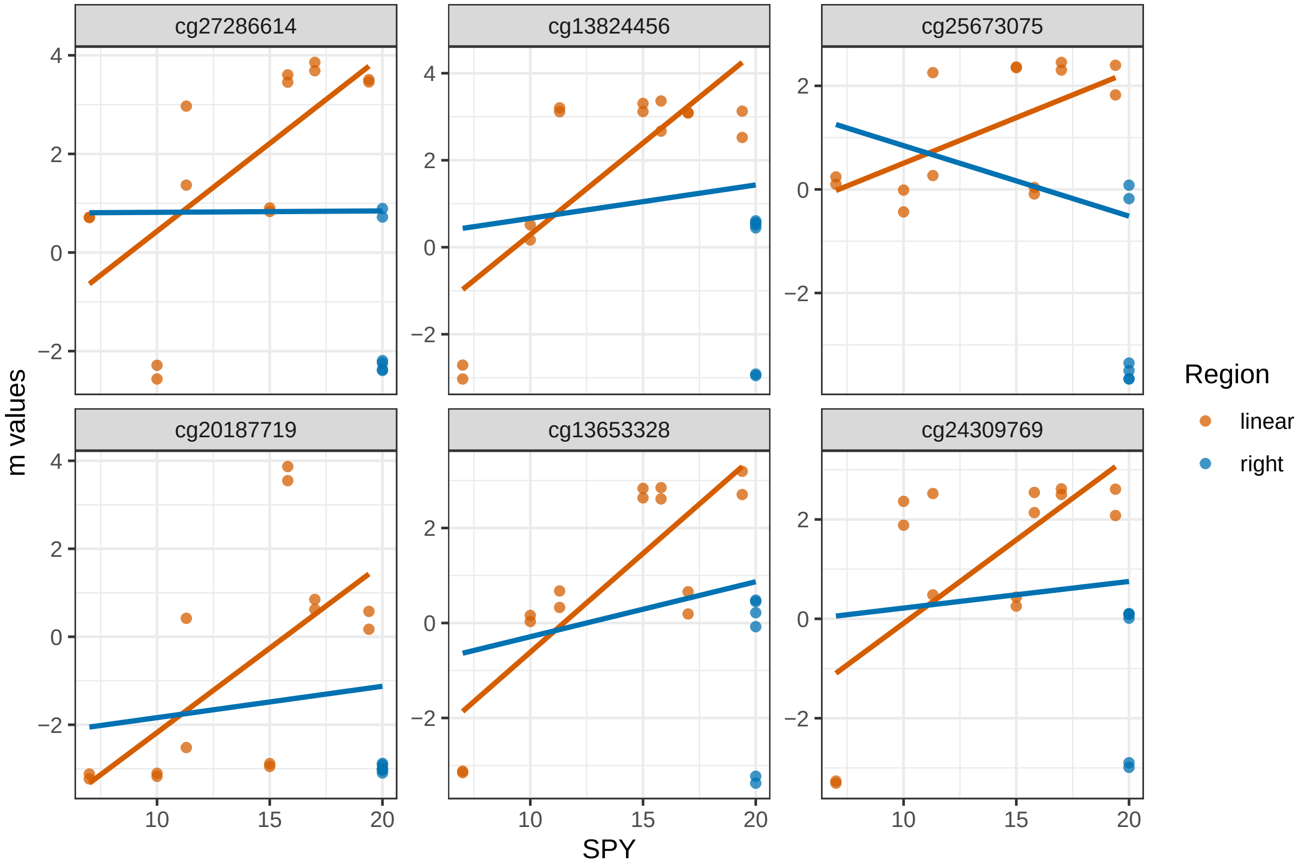

Supplementary Figure 2: Negation of the positive linear effect by the spike at right in the sample E-GEOD-54643.

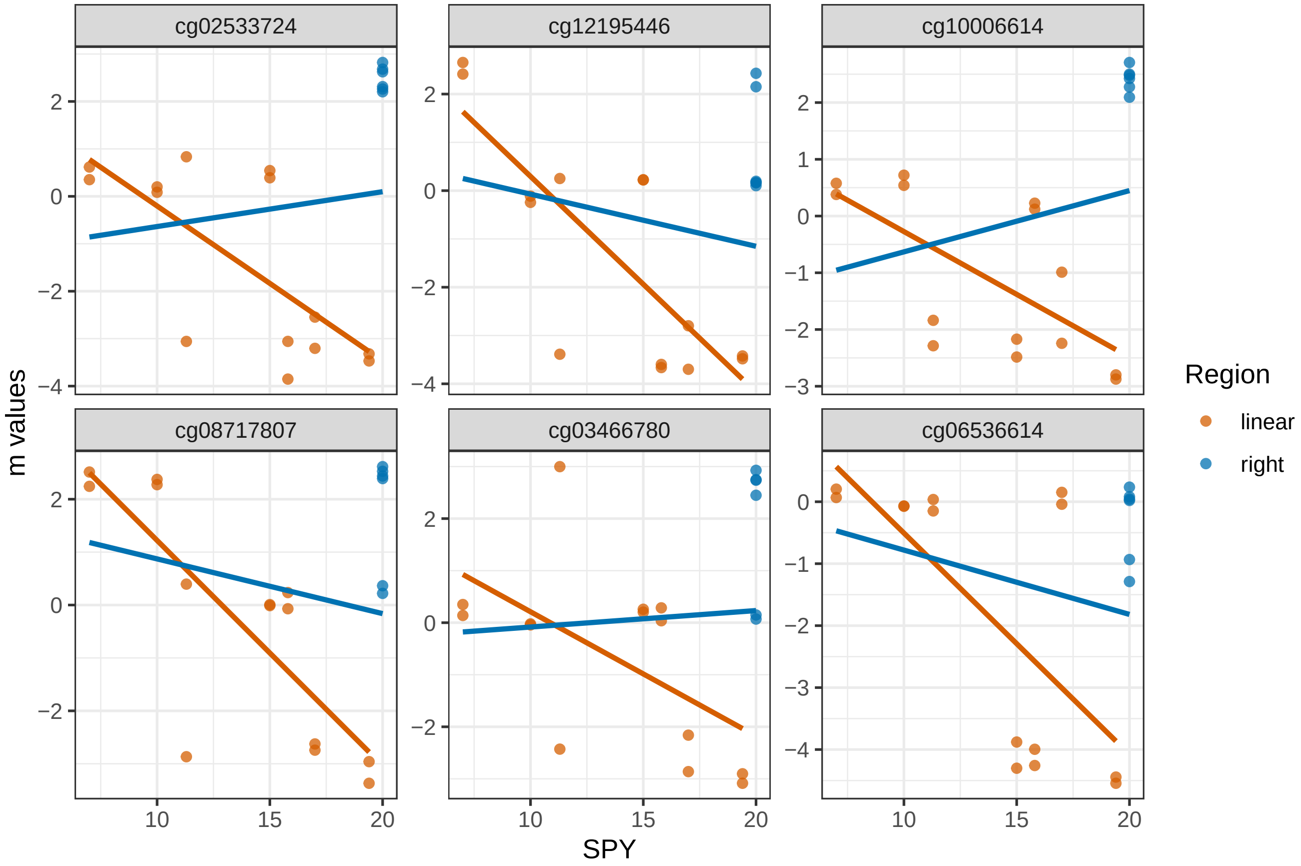

Supplementary Figure 3: Negation of the negative linear effect by the spike at right in the sample E-GEOD-54643

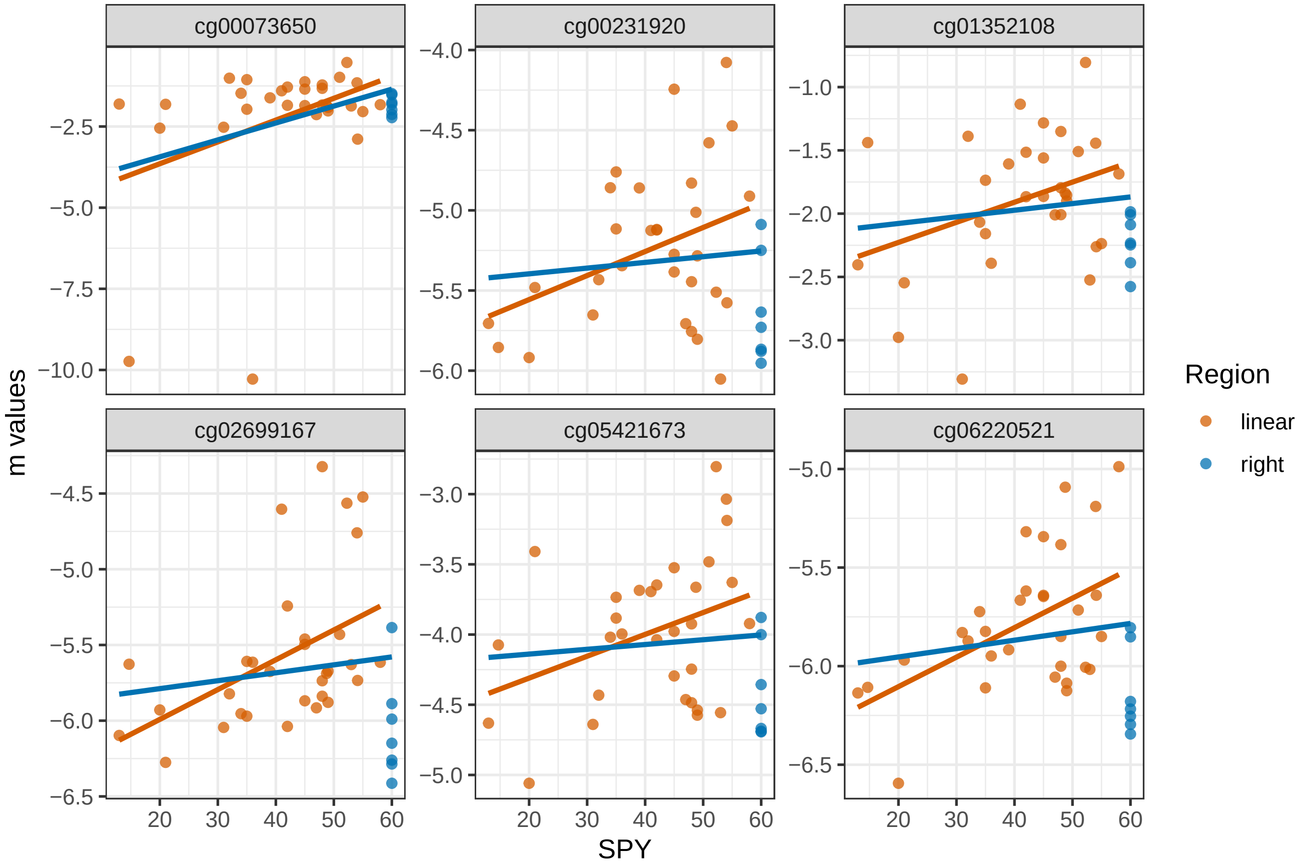

Supplementary Figure 4: Negation of the positive linear effect by the spike at right in the sample E-GEOD-55454

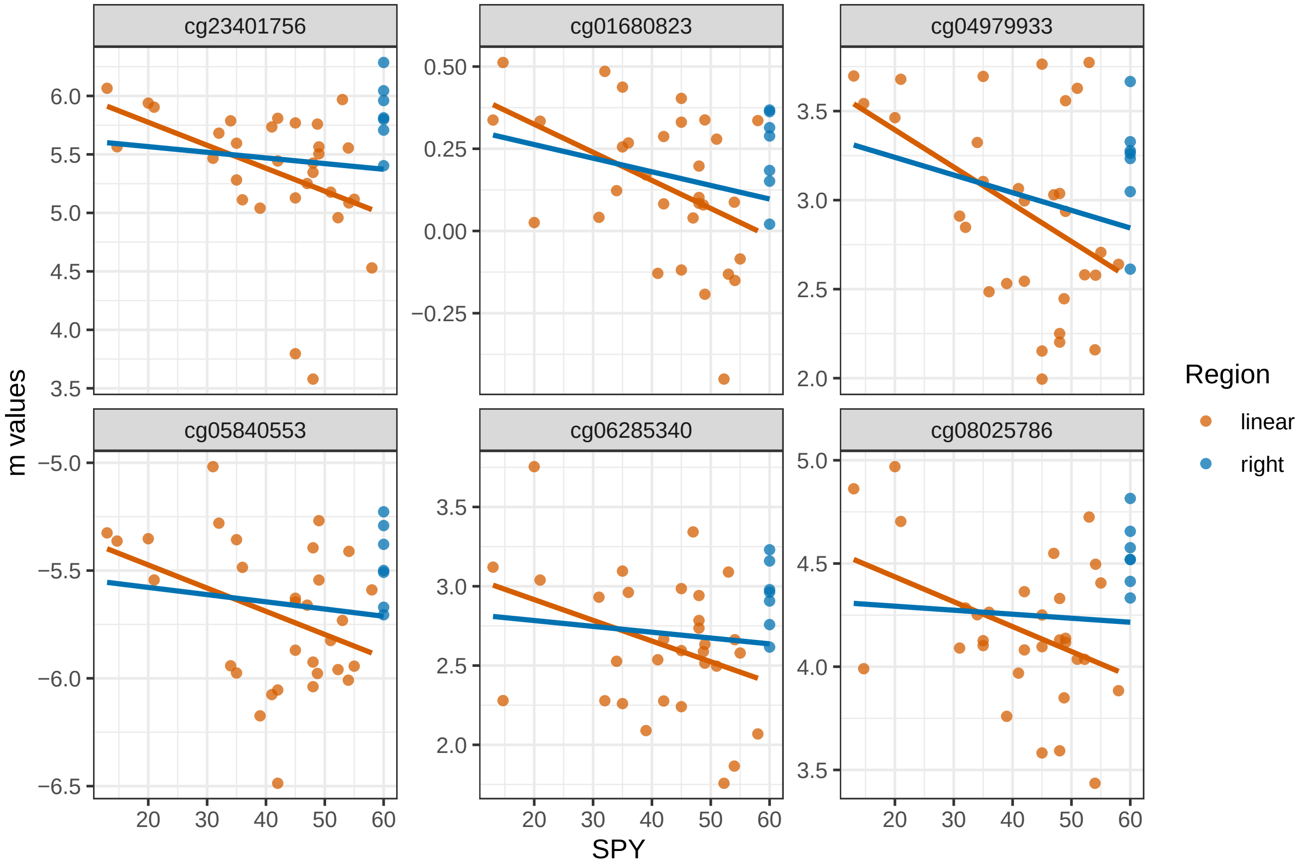

Supplementary Figure 5: Negation of the negative linear effect by the spike at right in the sample E-GEOD-55454

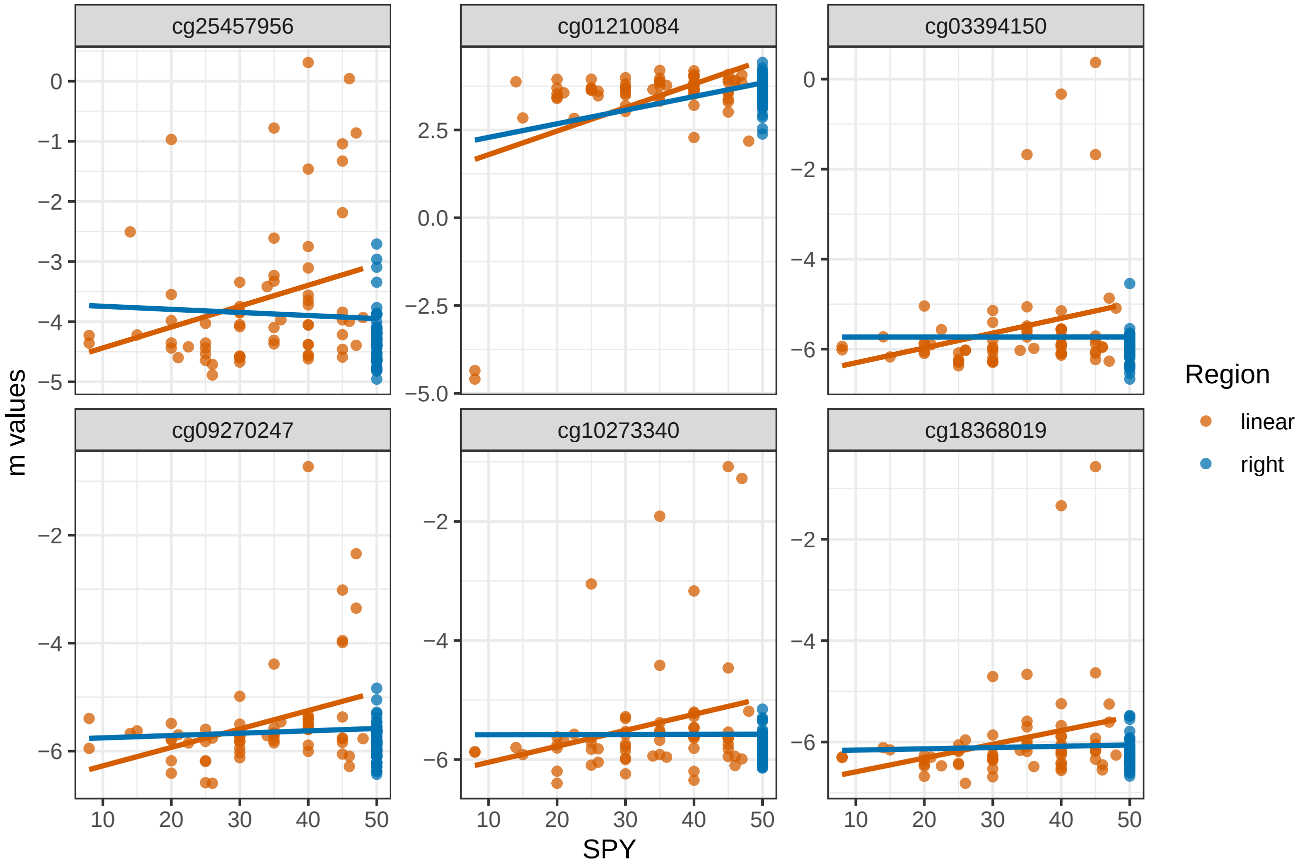

Supplementary Figure 6: Negation of the positive linear effect by the spike at right in the sample E-GEOD-68825

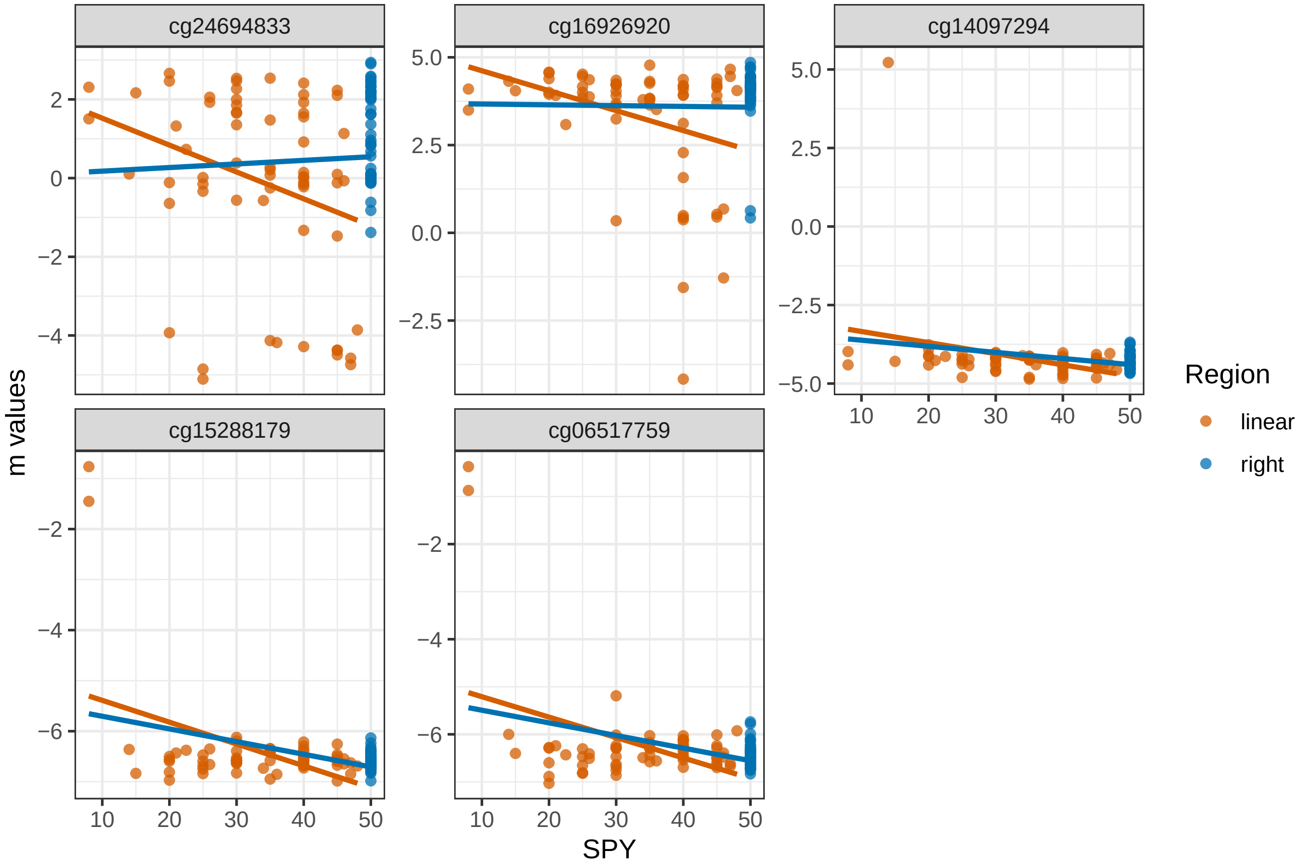

Supplementary Figure 7: Negation of the negative linear effect by the spike at right in the sample E-GEOD-68825

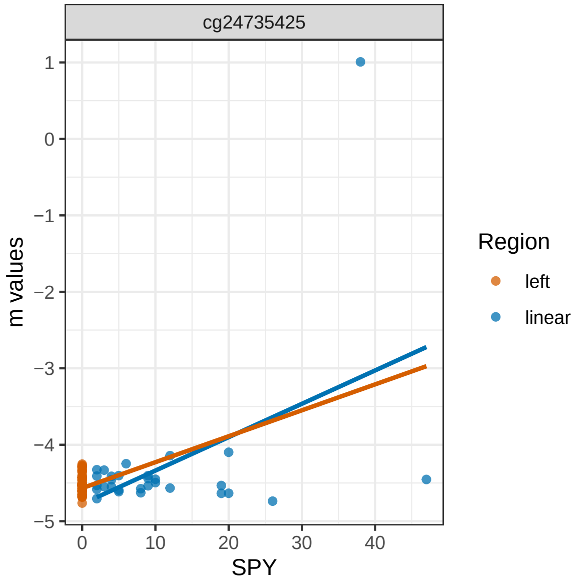

Supplementary Figure 8: Negation of the positive linear effect by the spike at left in the sample of Richter *et al.* (2019)[1]

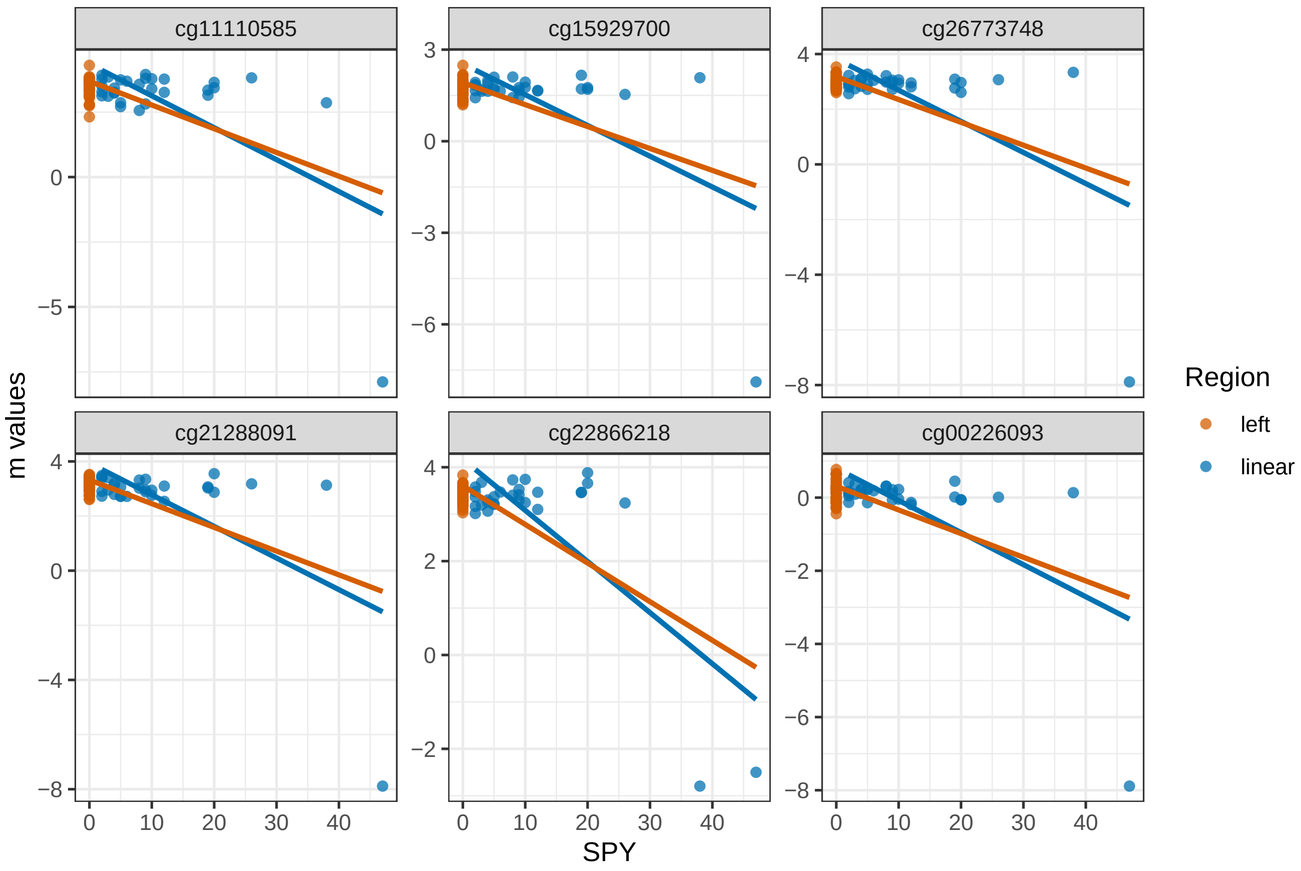

Supplementary Figure 9: Negation of the negative linear effect by the spike at left in the sample of Richter *et al.* (2019)[1]

References

[1] Gesa M Richter, Jochen Kruppa, Matthias Munz, Ricarda Wiehe, Robert Häsler, Andre Franke, Orlando Martins, Yvonne Jockel-Schneider, Corinna Bruckmann, Henrik Dommisch, et al. A combined epigenome-and transcriptome-wide association study of the oral masticatory mucosa assigns cyp1b1 a central role for epithelial health in smokers. *Clinical Epigenetics*, 11(1):105, 2019.
